# Pattern transitions in diary data of MDD patients: a mixed-methods multiple case study of psychotherapy dynamics

**DOI:** 10.3389/fpsyg.2024.1259610

**Published:** 2024-05-28

**Authors:** Sontje Nordholt, Philipp Garrison, Wolfgang Aichhorn, Matthias Ochs, Günter Schiepek

**Affiliations:** ^1^Department of Clinical Psychology, University of Osnabrück, Osnabrück, Germany; ^2^Department of Social Work, Fulda University of Applied Sciences, Fulda, Germany; ^3^University Hospital of Psychiatry, Psychotherapy, and Psychosomatics, Paracelsus Medical University, Salzburg, Austria; ^4^Institute of Synergetics and Psychotherapy Research, Paracelsus Medical University, Salzburg, Austria; ^5^Department of Psychology and Educational Sciences, Ludwig-Maximilian University, Munich, Germany

**Keywords:** mixed-methods approach, mixed grounded theory, change dynamics in psychotherapy, pattern transitions, process monitoring, diary texts

## Abstract

**Aim:**

Mixed-methods approaches promise a deep understanding of psychotherapeutic processes. This study uses qualitative and quantitative data from daily diary entries and daily self-assessments during inpatient treatment. The aim of the study is to get an insight into the similarities and differences between both types of data and how they represent self-organized pattern transitions in psychotherapy. While a complete correlation of results is not expected, we anticipate observing amplifying and subsidiary patterns from both perspectives.

**Materials and methods:**

Daily, five MDD patients wrote diaries and completed self-assessments using the Therapy Process Questionnaire, a questionnaire for monitoring the change dynamics of psychotherapy. The data were collected using the Synergetic Navigation System, an online tool for real-time monitoring. Diary entries of the patients described their experiences in everyday life. The qualitative text analysis was conducted using Mixed Grounded Theory, which provided categories representing the patients’ ongoing experiences of transformation and stagnation. The time series data was analyzed using the dynamic complexity algorithm and the pattern transition detection algorithm. Results from qualitative and quantitative analyses were combined and compared. Following the process of data triangulation, the leading perspective came from the theory of self-organization. In addition to presenting the overall results for all five patients, we delve into two specific case examples in greater detail.

**Results:**

Specific and highly diversified diary entries of 5 patients were classified into the categories of perceived pattern stability, noticing improvement, broadening the perspective, critical instability, and experiencing moments of Kairos. Patients reported problems not only related to their disorder (e.g., lack of energy and hopelessness) but also to phases and steps of change, which could be related to the theory of self-organization (e.g., problem attractors, critical fluctuations, pattern transitions, and Kairos). Qualitative and quantitative analysis provide important supplementary results without being redundant or identical.

**Conclusion:**

Data triangulation allows for a comprehensive and multi-perspective understanding of therapeutic change dynamics. The different topics expressed in the diary entries especially help to follow micro-psychological processes, which are far from being a simple reaction to interventions. The way patients experience themselves being in stability or instability and stagnation or transformation is surprisingly close to the general features of self-organizing processes in complex systems.

## 1 Introduction

The complexity approach in psychotherapy research conceptualizes patients as dynamic bio-psycho-social systems (Schneider et al., 1997; Strunk and Schiepek, 2006; Schiepek et al., 2017). Human systems create dynamic patterns of cognitions, emotions, and behavior by self-organizing processes (Olthof et al., 2020a). Pattern formation reduces the degrees of freedom for the overall system and its constituent parts and subsystems. Dynamic patterns that exhibit at least temporary stability and re-establish their dynamic features after perturbation are called attractors (Kriz, 1997; Haken and Schiepek, 2010). Pathological attractors, in general, may be seen as oversynchronized, rigid, and stable—lacking flexibility and adaptivity (Kashdan and Rottenberg, 2010; Tass and Popovych, 2012; Schiepek et al., 2021). Similar to many psychological and neurological disorders (e.g., addictions, obsessive-compulsive disorder, tinnitus, and Parkinsonian disease), depression may also be seen as a rigid bio-psycho-social attractor. Changes in attractor states are realized by discontinuous (non-stationary) transitions, as was shown in psychotherapy of depression and mood disorders in general (Lutz et al., 2009, 2013; Uher et al., 2010; Aderka et al., 2012; Schiepek et al., 2014; Helmich et al., 2020; Olthof et al., 2020a).

Therapeutic change processes usually realize order transitions from pathological patterns (attractors) to healthy patterns (Haken and Schiepek, 2010; Strunk and Schiepek, 2014). Critical instabilities often precede these order transitions, indicating that a former attractor gets destabilized and a new one emerges. These change-sensitive moments may be experienced as periods of “Kairos,” which refers to the right moment for therapeutic intervention (Strunk and Schiepek, 2006; Haken and Schiepek, 2010). Olthof et al. (2020a,b) identified critical instabilities by increased dynamic complexity in time series as a short-term predictor of order transitions in patients with mood disorders. In a non-clinical sample, Van De Leemput et al. (2014) found a critical slowing down in the dynamics of emotions associated with an increased risk of a subsequent shift to clinically significant depression. In the same study, a sample of patients with depression showed a positive correlation between critical slowing down in emotional dynamics and the likelihood of recovery. In a case study, Wichers and Groot (2016) observed a critical slowing down associated with a subsequent sudden change in depressive symptoms.

To investigate psychotherapeutic change from a dynamic systems perspective, quantitative, qualitative, and mixed-methods approaches were applied (Gelo et al., 2012; Gelo and Manzo, 2015; Schiepek, 2020). Qualitative methods were used with reference to the paradigm of self-organization by Beirle and Schiepek (2002), who coded 13 and 9 video-taped sessions of two brief solution-focused psychotherapies applying configuration analysis (Horowitz, 1987). They identified the states of mind of the patients as they occurred during the sessions. The analysis of the video tapes revealed that specific states, highlighted by patients as significant during the sessions, did not manifest within those sessions. The mapping of the change process relied on video tapes but did not incorporate data from real-world settings.

Another qualitative approach to human change dynamics was realized by Gennaro et al. (2020). The authors applied automated text analysis (Automated Co-occurrence Analysis for Semantic Mapping) to dream narratives reported during psychoanalytic therapy (in 95 out of 517 sessions, dreams were reported). The dream narratives showed an increased intensity of affective charges and an increased frequency of affective-laden meanings. Furthermore, a non-linear phase transition in the complexity of affective dream dynamics within the phase space of the meaning dimensions was identified. Similar methods were applied to another set of dream narratives by Mariani et al. (2021).

Gonçalves et al. (2011) developed the Innovative Moments Coding System (IMCS) to identify changes and emerging topics within psychotherapeutic processes. The IMCS can be applied to qualitative text material from therapy sessions or to other discourse or conversational data. Innovative moments refer to changed functionalities in dealing with different issues rather than to the contents that are discussed in psychotherapy (Fernández-Navarro et al., 2020). Gonçalves et al. (2011) shared qualitative similarities with self-organized pattern transitions and defined five types of innovative moments: action, reflection, protest, reconceptualization, and performing change, with each category having different impacts on the transformation process. The IMCS has been applied in studies on process data of patients diagnosed with depression (Cunha et al., 2012; Gonçalves et al., 2017; Fernández-Navarro et al., 2018). An exploratory study by Gonçalves et al. (2017) provided evidence that the occurrence of innovative moments in a therapy session predicts reduced depressive symptoms in the following session.

The studies reported have focused on change processes during therapy sessions. In addition to the sessions, patients have important experiences in their real-word settings. Consequently, diary surveys are useful for tracing individual transformation processes in everyday experiences. Diaries capture events close to their actual occurence (every day) and allow subjective assessments, so the diary method seems appropriate to map individual change trajectories over a longer period of time (Lundgren et al., 2018). Diary studies were used for a comparison between depressed subjects and non-clinical samples (Molendijk et al., 2010; Baddeley et al., 2013; Krejtz et al., 2020). Beyond research and data acquisition, writing diaries may have therapeutic effects (Suhr et al., 2017).

Up to now, there are only a few diary studies on individualized change processes of patients diagnosed with depression. Lundgren et al. (2018) used a diary method to map mindfulness-based training of patients with heart disease and depressive symptoms. Using qualitative content analysis, they identified two categories (“facing the challenges of daily practice” and “harvesting the fruits of daily practice”) for mapping trajectories of change. The first category occurred frequently in the first 2 weeks of the training program, and the second category more frequently during 3 to 8 weeks. In two single case studies from our group, diary texts were used to illustrate subjective experiences during pattern transitions, identified using quantitative time series analysis (Schiepek G. K. et al., 2016; Schiepek et al., 2023).

Diaries capture a comprehensive picture of a patient’s everyday life compared to data from therapy sessions only. However, there are only a few diary studies on psychotherapeutic change processes within the paradigm of self-organization. The aim of this study is two-fold: First, it aims to gain insight into the narratives of change from a perspective of self-organization. Second, the study aims to contribute subjective meaning to quantitative assessments by integrating qualitative and quantitative data. By employing a mixed-methods design that incorporates diary data and quantitative time series data, the study seeks to enhance ecological validity by providing a holistic and multi-dimensional perspective on psychotherapeutic processes (Ochs, 2012; Bartholomew and Lockard, 2018; Bailey-Rodriguez, 2021).

Given this background, with the following two open questions, we are investigating our research interest:

1. How are psychotherapeutic change dynamics represented in diary entries of patients diagnosed with depression and does this align with the assumptions of self-organization?
2. How are indicators of self-organized dynamics in the diaries and in time series data related to each other?

By relating time series data and diary data, the present study tries to link a nomothetic perspective on self-organized pattern transitions with the idiographic perspective of patients’ subjective experiences to contribute to a deeper understanding of the therapeutic journey. Relating the two perspectives may also enable the transfer of research findings to clinical practice.

## 2 Materials and methods

### 2.1 Participants

The sample of 5 patients (3 women, 2 men, mean age 38.8 years with a range from 26 to 50, and diagnosis: major depressive disorder) was selected from a pool of existing data that was collected during the clinical routine practice at the inpatient psychotherapy center of the University Hospital of Psychiatry, Psychotherapy, and Psychosomatics (Paracelsus Medical University, Salzburg). Patients used the Therapy Process Questionnaire (TPQ, Schiepek et al., 2019) for daily self-assessments and wrote a diary entry at the end of each day. Data were collected using the Synergetic Navigation System (SNS), an online and app-based tool for real-time monitoring of psychotherapy processes, which also allows non-linear analysis of time series data (Schiepek et al., 2018a).

One criterion for selecting cases was a sufficiently large text body, defined as a minimum of 50 words per diary entry. Therapists who were familiar with the patients were consulted to evaluate the data quality of the selected cases. The time series should have a length of at least 60 measurement points (= days), and there should be less than 10% missing data in the time series. As a requirement of the Mixed Grounded Theory method (Strauss, 1998; Mey and Mruck, 2011; Truschkat et al., 2011; Truschkat, 2013), the principle of contrasting asks for cases that differ in age, gender, and length of the text corpus (over vs. under 4,500 words). Another criterion of case selection was the existence of a distinct pattern transition in the time series, which was identified by the pattern transition detection algorithm (Viol et al., 2022).

Patients signed an informed consent form for the procedure of using the SNS during their hospital stay. The study was performed in accordance with the declaration of Helsinki and was approved by the ethics committee of Salzburg County (415-E/1068/3-2009). The diary data were anonymized, and pseudonyms were used to refer to specific persons in the narrative. In addition to providing general results based on the sample of five patients, we present detailed accounts of two cases (male patients) to exemplify the interconnectedness between qualitative and quantitative data.

### 2.2 Measures

We chose a multiple case study approach (e.g., Barela, 2007; Stake, 2013) to explore our research questions. Given our objective to construct a detailed and multi-faceted understanding of psychotherapeutic change processes, we selected a mixed-methods multiple case study design (e.g., Vanderkerken et al., 2019). This approach allows for the inclusion of diverse perspectives on the subject through data triangulation (Völcker, 2019; Völcker et al., 2019). Following mixed-methods data analysis, we integrate both quantitative and qualitative data sources (Plano Clark et al., 2008). Our design involved utilizing quantitative and qualitative therapy process data obtained from the Synergetic Navigation System (Schiepek et al., 2018a).

For the quantitative analysis, we employed well-established methods aligned with self-organization theory to measure stability and transformation. On the qualitative front, we analyzed diary data using the Grounded Theory Methodology. Grounded Theory allows us to respond flexibly to the data and to develop a data-driven theoretical conceptualization rather than imposing a predefined system of categories (see for integration of Systems Theory and Grounded Theory, e.g., Levina, 2021).

While all five subjects’ data contributed to the development of transformation indicators, we used data from two cases to illustrate the results.

The Therapy Process Questionnaire (TPQ, Schiepek et al., 2019) was used for daily assessments. It comprises 43 items assigned to 7 factors: “Well-being and positive emotions” (WPE), “relationship with fellow patients” (RFP), “therapeutic alliance and clinical setting” (TAS), “emotional and problem intensity” (EPI), “insight/confidence/therapeutic progress” (ICP), “motivation for change” (MOT), and “mindfulness/self-care” (MSC). The items were scored using Visual Analog Scales, which were numerically transformed to values from 0 to 100. In addition to the quantitative self-assessments, the patients used the comment function of the SNS to write daily diary entries (free format).

The time series sample ranged from 76 to 99 measurement points, with an average of 84 words per diary entry. On average, each patient’s diary comprised 7,346 words. There was an average of 1.6% missing values in the quantitative data and 15.20% missing entries in the qualitative data. Missing values in the time series were replaced by the value of the previous day (the last observation carried forward). The higher rate of missing diary entries compared with the quantitative self-assessments may be explained by the fact that writing diaries is more time-consuming than filling in the questionnaire. Additionally, the time series diagrams received continuous attention during therapy sessions, while writing diaries was considered optional. Patients were informed that both the time series and diaries could be reviewed by their therapists, suggesting that social desirability and other communicative biases cannot be entirely ruled out.

### 2.3 Time series analysis

The dynamic complexity measure (DC; Haken and Schiepek, 2010; Schiepek and Strunk, 2010) identifies critical instabilities in real-world time series without specific statistical or parametric assumptions. It mirrors the increased complexity and sensitivity to noise and perturbations of system dynamics before a critical event or a phase transition occurs. DC is the multiplicative product of a fluctuation measure (F) and a distribution measure (D) applied to discrete time series data with given data ranges and constant discrete time intervals between the data points. F is sensitive to the amplitudes and frequencies of a time signal, and D scans the scattering of values or system states within the range of possible values. To identify non-stationarity, DC is calculated within a data window sliding along the time series (window width: 7 measurement points). To identify significant periods of critical instabilities in the time series, we realized surrogate testing by comparing the empirical results to multiple random time series generated from the original values in a randomized sequence (Haken and Schiepek, 2010).

The pattern transition detection algorithm (PTDA, Schiepek et al., 2020; Viol et al., 2022) was developed to detect pattern transitions in time series data. The method applies change point analysis (CPA, Killick et al., 2012) to the empirical time series and to the “second level” time series as the DC measure, Recurrence Plots, and Time–Frequency Distributions (TFDs). Six change points can be identified: one each for changes in the mean, the variance, and the linear trend of the empirical time series, and one for a change in the mean of the DC, the lines of the Recurrence Plot (RP), and the frequency levels of the TFD. For this, the PTDA constructs Recurrence Plots and TFDs. Recurrence Plots (RP) provide visualization and quantification of recurrent, i.e., dynamically repeating state values within a time series (Marwan et al., 2007; Webber and Marwan, 2015). In the RP method, dynamic similarity is measured in terms of metric distances between vector points in a state space. Each vector point represents a small sequence (snippet) of measurement values, and all embedded vector points grasp the whole time series, which is projected into a multidimensional state space with a specific embedding dimension m and a time-delay τ. TFD is a method to calculate and visualize the frequency of a signal as it changes with time (Cohen, 1989). To identify frequency changes, a moving window approach is implemented. Both time t and frequency ω are variables of a distribution P (t,ω), which describes the amplitude (energy) of the signal at each given t and ω. Time and frequency are plotted on a plane (x: time, y: frequency), and color coding is used to represent the amplitude (energy) of the frequencies. In the RPs and TFDs, the CPA with respect to the changes in mean levels is applied to every line. Then, a histogram of the change points of all lines is built, and the peak of the histogram determines the point of change for the whole matrix. The probability of a pattern transition in a given process is based on all change points identified in the original time series and all “second order” measures. PTDA also uses surrogate testing for probability estimates of pattern transitions.

### 2.4 Qualitative data analysis

The qualitative text analysis was realized using the Mixed Grounded Theory, which allows the analysis of quantitative and qualitative data in parallel (Johnson and Walsh, 2019). Quantitative results were used for a theoretical orientation during the theoretical sampling process of the qualitative analysis. As a final step, we merged qualitative and quantitative results at the individual level. The process of data triangulation thus allowed the development of a detailed picture of the dynamics (Völcker, 2019; Völcker et al., 2019).

While Grounded Theory, as advocated by Glaser and Strauss, mandates the complete exclusion of prior knowledge to formulate a new theory solely grounded in the data (a strictly bottom-up procedure), our approach diverged by adopting the constructivist perspective, as proposed by Charmaz (2008). The Constructivist Grounded Theory states the impossibility of maintaining a neutral standpoint during data analysis, asserting that researchers are inevitably influenced by their prior knowledge—no researcher is a “tabula rasa” (Charmaz, 2000, 2008). Consequently, it frames the analysis process as an ongoing interaction between the researcher and the data, emphasizing continuous reflection on one’s reasoning before considering prior knowledge.

The data analysis was performed as part of the first author’s Master’s thesis, focusing on complex systems in psychology within a clinical psychology program. The analysis was part of an interdisciplinary project. Therefore, the data analyst had prior knowledge regarding psychological concepts, particularly the complex systems approach in psychology. However, we emphasize that the relevant prior knowledge should not decisively shape direct interpretations. To mitigate potential bias, the first author derived codes by thoroughly examining the diary data word-by-word. The guiding principle for assigning codes to the diary data was identifying indicators for stability and transformation in the text. Through an ongoing interdisciplinary dialogue between a psychologist (SN) and a sociologist (PG), we meticulously scrutinized and evaluated categories and text passages with fine granularity and continuity, ensuring that no single reading led to a predetermined result. This procedure is described by Steinke (2010) in her systematization of quality criteria in qualitative research as intersubjective confirmability. However, we acknowledged that an absolute exclusion of prior knowledge is not imperative nor feasible with the newer approaches of Grounded Theory. To further enrich our analysis, we later reflected on the developed categories by comparing them to concepts of self-organization theory (e.g., emergence of new topics, discontinuous pattern transitions, and critical instabilities before or during pattern transitions). This comparative analysis was instrumental in gaining deeper insights into the meanings associated with the identified categories.

In Grounded Theory, the coding process follows an iterative-cyclic procedure of open, axial, and selective coding. During the coding process, the three coding phases are not entered in a linear manner but in an iterative-circular manner. Combined with the principle of minimum and maximum contrasting, the codes were continuously checked and concretized during the process.

**Figure 1:**
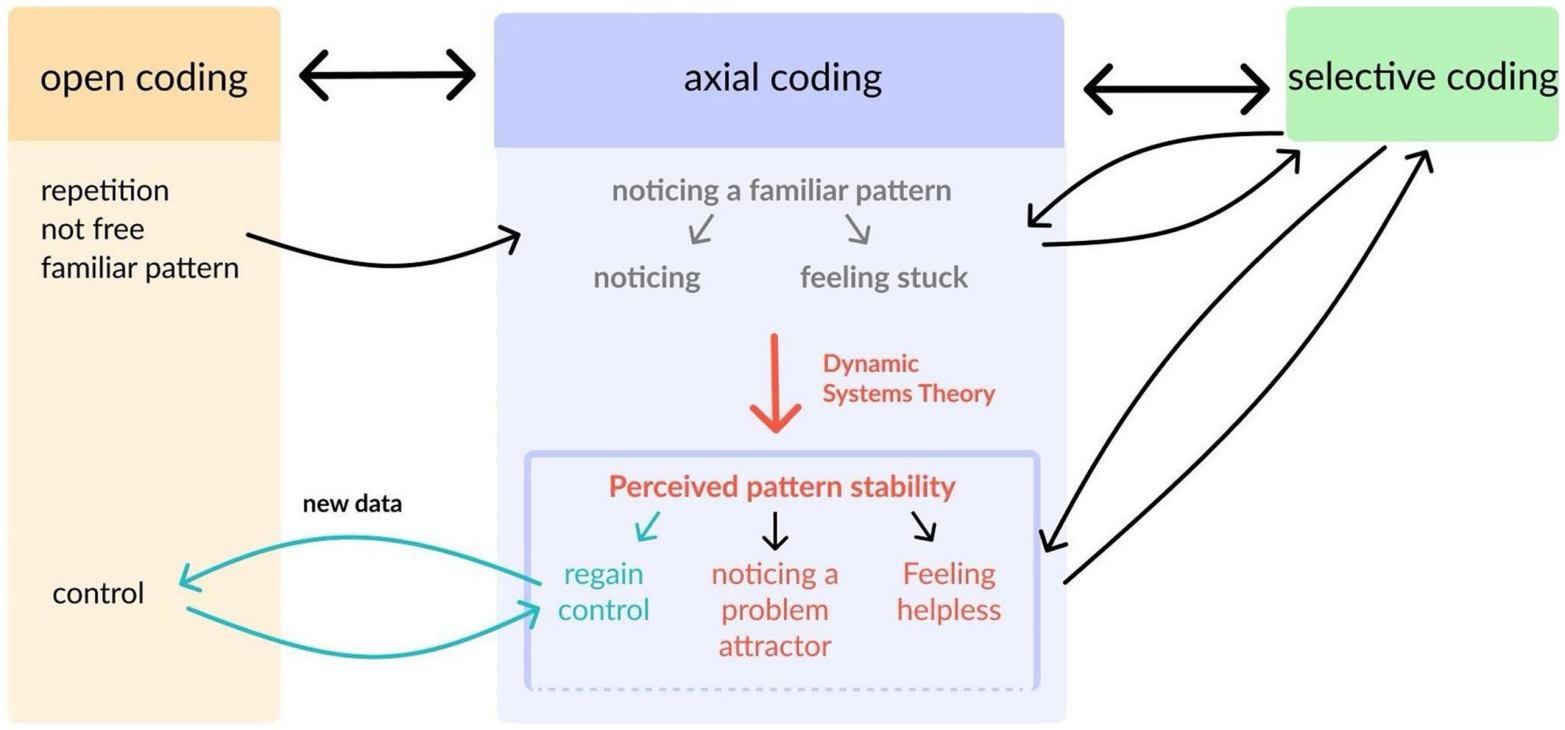
The process of open, axial, and selective coding illustrated using the example category of “perceived pattern stability”.

**Figure 2:**
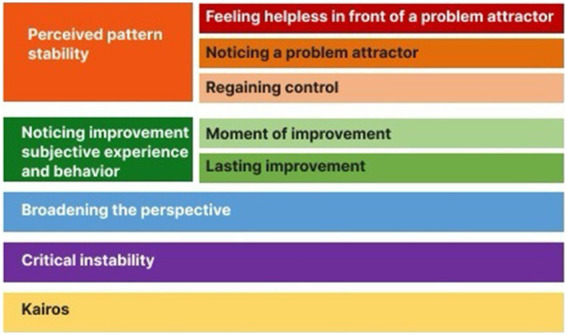
Overview of the indicators of transformation that resulted from the Grounded Theory analysis of the diary data of five patients.

Here, we illustrate the coding process and the integration of prior knowledge, focusing on the example category of “perceived pattern stability,” which is depicted in Figure 1. The conclusive model and definitions of categories can be found in the results section (see also Figure 2).

The coding process begins with an initial open coding phase. The data material undergoes thorough coding, ranging from line-by-line to even word-by-word analysis without constraints. The aim of this small-step procedure is to find initial provisional concepts that represent the data concerning the research question. Following de Felice et al. (2022), examples of open coding codes and corresponding text segments for all categories can be found in Table 1.

**Table 1:** Examples of illustrative text segments for initial open coding codes.

Code	Text segment
Repetition	Leider habe ich wieder einmal mehr meine Grenzen nicht geachtet, ich habe abermals eine Zurückweisung von meiner Exfreundin erhalten, sie hat für ihr Verhalten jedes mal eine Begründung, auch wenn ich dadurch verletzt werde. *Unfortunately, once again I have not respected my boundaries, I have once again received a rejection from my ex-girlfriend, she has a reason for her behaviour every time, even if it hurts me.*
Unfree	..ich habe das Gefühl, dass ich nicht weiter komme, ich fühle mich unfrei, eingeschränkt, abhängig…! *..I have the feeling that I’m not getting anywhere, I feel unfree, restricted, dependent…!*
Familiar Pattern	Da realisierte ich auch wieder einmal, wie sehr ich durch solche äußere Einflüsse wie die Stimmung in der Gesellschaft, politische Strömungen etc. in meiner Stimmung (mit-)beeinflusst bin. *I realised once again how much my mood is influenced by external influences such as the mood in society, political currents,* etc.
Breaking patterns through alternative behavior	Es gelingt mir offenbar gerade sehr gut, mich nicht von etwaig auftauchenden “negativen” gefühlen mitreißen zu lassen bzw. Mich gegen sie zu wehren. *I seem to be doing very well at the moment in not letting myself be carried away by any “negative” feelings that may arise or in resisting them.*
New self-realization	Konnte etwas neues aus der Achtsamkeitsgruppe mitnehmen, das ich so noch nicht in meinem Bewusstsein hatte. *I was able to take something new away from the mindfulness group that I had not yet realised.*
Inner process	Im Großen und Ganzen habe ich das Gefühl das einige Dinge in mir arbeiten und ich hoffe, dass ich die richtigen Türen offenhalten kann um nachhaltig etwas in meinem Erleben zu verändern. *Overall, I have the feeling that some things are working in me and I hope that I can keep the right doors open to make a lasting change in my experience.*
It simply happened	In der Einzeltherapie “passierte” mehr etwas, als dass ich es bewusst gemacht hätte, und dennoch ist mir hinterher bewusst geworden, dass das ein ziemlich großer Schritt für mich war. *In the individual therapy, something “happened” more than I consciously did it, and yet I realised afterwards that this was a pretty big step for me.*
Disoriented	Habe derzeit das Gefühl dass ich mich zurückziehen möchte…Verspüre eine aufkommende große Trauer in mir….bin teilweise irgendwie wie ‘orientierungslos’. Versuche mich ganz bewußt wieder zu ankern. *At the moment I have the feeling that I want to withdraw… I feel a great sadness rising inside me…. Trying to consciously anchor myself again.*
“Lost the thread”	Diffus müde, unmotiviert, als ob “ich den Faden verloren hätte,” großer innerer Widerwillen. *Diffusely tired, unmotivated, as if “I’ve lost the thread,” great inner reluctance.*

For instance, excerpts such as: “Ich fand das insgesamt sehr unnötig, **konnte** aber, **wie** es bei mir **immer** ist in so einer geladenen Situation, **nichts dazu sagen**.” *(Overall, I found it very unnecessary, but **I couldn’t** say anything, **as always** in an emotionally charged situation like this) or “*Am Abend habe ich mich noch mit einem Kollegen getroffen. Es war ganz nett, aber irgendwie hatte ich **schon wieder** dieses Gefühl mit den Menschen **nicht richtig reden zu können**.” (*I met a colleague in the evening. It was fine, but **once again** I somehow had this feeling that **I could not** really talk to people*) led into *in-vivo* codes, such as “wie immer” (as always), “wieder mal”/“wieder einmal”/“abermals” (once again), denoting expressions of repetition when the subjects described their own behavior. Additionally, codes like “unfrei” (not free) were assigned to phrases such as “ich konnte nicht anders” (I could not do otherwise) or “ich musste” (I had to), while “bekanntes Muster” (familiar pattern) was applied when subjects recognized their behavior as a recurrent pattern (see Figure 1). These codes could be assigned to text passages of all five cases.

Moving on to axial coding, a specific category undergoes thorough analysis to increase the knowledge about this category. This process reveals relationships between different categories and allows for the development of subcategories (Strauss, 1998).

In our case, we consolidated the codes that were mentioned above into the category “noticing a familiar pattern.” By examining the codes assigned to this category, we defined this category as the reporting of an unwanted and familiar pattern, marked by indicators such as repetitive phrases, perceived lack of freedom, and self-identification of behavior as a pattern often accompanied by frustration or regret. The subcategories “noticing” and “feeling stuck” were introduced to describe variance within the category (see Figure 1).

During the selective coding phase, we revisited the text data and applied the preliminary categories to gain—under this perspective—further information about the concepts and the data. The category “noticing a familiar pattern” evolved further during another axial coding process. Here, we incorporated the dynamic systems theory, highlighted in orange in Figure 1. We chose the term “problem attractor” to specify the category “noticing a known pattern” since the definition of the category aligns with the characteristics of an attractor: a relatively stable structure with few degrees of freedom, a tendency to revert to these patterns when perturbed, and centrality in psychotherapy as targets for change (Kriz and von Schlippe, 2011; Hayes and Yasinski, 2015).

However, following axial coding or selective coding, it is not uncommon to revisit the open coding phase, particularly when incorporating new data into the analysis. In our example, incorporating new data material added a new facet of noticing pattern stability that was later described by the subcategory “regaining control,” highlighted in blue in Figure 1.

Now termed “perceived pattern stability,” this category was therefore finally subdivided into three nuanced subcategories: “feeling helpless in front of a problem attractor,” “noticing a problem attractor,” and “regaining control,” to explain further variance in the text data, each elucidated in the results section. Examples of corresponding text segments for the subcategories of all indicators for transformation are provided in Table 2.

**Table 2:** Examples of text segments assigned to each subcategory.

Category	Original text segment
Feeling helpless in front of a problem attractor	Ich stecke irgendwie fest in dem Abhängigkeitsgefühl, ich spüre, dass ich mein Wohlbefinden/ Glücksgefühl von einer Reaktion meiner Exfreundin abhängig mache, leider! *I am somehow stuck in this feeling of dependency, I sense that I make my well-being/happiness dependent on a reaction of my ex girlfriend, unfortunately!*
Noticing a problem attractor	Am Abend habe ich mich noch mit einem Kollegen getroffen. Es war ganz nett, aber irgendwie hatte ich schon wieder dieses Gefühl mit den Menschen nicht richtig reden zu können. Als ob in meinem Kopf Löcher wären, wo ich mein Wissen nicht mehr abrufen kann und dann fühle ich mich dumm… *I met a colleague in the evening. It was fine, but once again I somehow had this feeling that I could not really talk to people. As if there were holes in my brain, where I’m unable to retrieve my knowledge and then I feel stupid…*
Regaining control	Ich habe aber auch eine Nachricht von einer Freundin erhalten, die mich sehr verunsichert hat und in Verlegenheit gebracht hat. Im Nachhinein war es ein Scherz, aber für mich war es eine große Enttäuschung und gleichzeitig wieder eine Bestätigung, dass ich mich auf niemand anderem verlassen kann, außer mir selbst. Auf der einen Seite war ich sehr enttäuscht, aber auf der anderen Seite habe ich mir einfach gedacht, dass ich mich über ein so kindisches Verhalten nicht ärgern werde. *But I also received a message from a friend that made me very insecure and embarrassed me. In hindsight, it was a joke, but for me it was a big disappointment and at the same time again confirmation that I cannot rely on anyone else but myself. On the one hand, I was very disappointed, but on the other hand I just thought to myself that I wasn’t going to get angry at such childish behaviour.*
Moment of improvement	„Ich schaff es grad ganz gut nicht zumindest kurz ins Bett zu fallen, sobald ich heimkomme, was ich schon immer als kurzes Entfliehen aus der Realität empfand“ *Currently, I manage not to fall into bed as soon as I come home, which I always perceived as a brief escape from reality.*
Lasting improvement	„es hat sich nun doch etwas verändert. Ich bin wieder fühlbar mehr in der ‘Realität’…auch wenn ich oftmals noch eine gewisse Distanz und auch Entfremdung wahrnehme..“ *Now something has actually changed. I‘m palpably more in “reality” again… although I often still sense a certain distance or alienation.*
Broadening the perspective	Ich durchlebte quasi eine schmerzhafte Situation erneut - dieser “Fehler” wird mir in letzter Zeit öfter bewusst, nämlich dass ich mich gedanklich sehr viel in der Vergangenheit aufhalte und dabei vornehmlich negative Emotionen mit in die Gegenwart nehme. Die positiven gehen dabei irgendwie unter. *I was reliving a painful situation, so to speak - I’ve been realising this “mistake” more often recently, namely that I spend a lot of time dwelling on the past in my thoughts and mainly take negative emotions with me into the present. The positive ones somehow get lost in the process.*
Critical Instability	Es war mir alles so vertraut und doch so fremd…Ein Gefühl was mich zu zerreißen drohte…Ich bekam Angst…Angst wie es mit mir beruflich weitergehen soll…ICH BIN ICH und doch bin ich es nicht mehr…Ich fühle mich so verloren ohne Halt,ohne Wurzeln, ohne gefühlte Vergangenheit… *It was all so familiar and yet so strange…A feeling that threatened to rip me apart… I became frightened…afraid of how I will proceed professionally…I AM ME and yet I am not me anymore…I feel so lost and without stability, without roots, without a past, that I can feel…*
Kairos	- Als wie wenn es von irgendwem geplant worden wäre, ist mir heute in der Psychodrama-Gruppe genau das Thema begegnet, das mich derzeit sowieso beschäftigt - mich abzugrenzen[…]. Vielleicht bin ich aber auch nur besonders auf dieses Thema angesprungen, weil meine Wahrnehmung da grad etwas geschärft ist - ist aber auch egal, tut nichts zur Sache. Auf jeden Fall hat mich diese Konfrontation damit heute zusammen mit den Erfahrungen und Gedanken der Vortage sehr zuversichtlich gestimmt, da zunehmend etwas zu “verstehen” und es mir dadurch ermöglicht wird, mehr Aufmerksamkeit in diese Richtung zu lenken und dadurch etwas zu verändern - oder auch nur bewusster zu machen.” *As if it was planned by somebody I came across the exact topic I was occupying myself with lately anyways – setting boundaries[…]. Maybe I immediately reacted to that topic, because my perception is heightened in that direction right now – anyways, it doesn‘t really matter.* *I any case, this confrontation today in combination with the experiences and thoughts of the preceding days made me somewhat confident to increasingly “understand” something there and that makes it possible for me to direct my attention in that direction and to change something by that – or at least to become more aware of it.*

In the final phase of individual case evaluation, a comprehensive comparison was conducted between the outcomes of both quantitative and qualitative analyses within the individual processes. Specifically, segments of time featuring qualitative indicators of transformation were scrutinized to determine whether these instances were mirrored at a quantitative level, manifested through critical instability or order transitions. Conversely, periods where quantitative data revealed order transitions or critical instability were examined in the qualitative data. Through this correlation, distinctions and resemblances between quantitative and qualitative transformation indicators were pinpointed, aiding in a nuanced understanding of these concepts.

## 3 Results

In each diary, we identified person-specific topics that occurred repeatedly. Assuming that the act of writing diaries is based on a process of reflection, patients decided which experiences were important to them and should be recorded. They reflected on these events in an ongoing process and identified changes in their own behavior and experiences related to their topics. The main categories of the identified topics were classified as “individual symptoms of depression,” “relationship to the self,” and “relationship to others.” Despite the shared diagnosis of depression, the diaries of the five patients showed a broad range of different topics. In four of the five cases, depressed mood and fatigue were shared topics. How to establish a daily routine, feelings of inner emptiness, and somatic symptoms were topics in three of five cases. The experienced relationship to the own self, self-esteem, and questions of identity development were topics for two out of five patients. The relationship with others was important in all cases, with a specific issue in how to protect one’s own self.

It should be noted that the change lies not in the content of the indicated topics but in how a person expresses them in writing. The indicators of change relate to the “how” of reporting on the topics, not so much to the content. We introduce the model we developed using Grounded Theory: Indicators of Transformation. For an overview of the indicators of transformation, see Figure 2.

### 3.1 Indicators of transformation: phases

The diaries revealed different phases or periods of dealing with personal issues and topics. The phases represent operations that patients apply to their own issues, providing information about mental processes during the transformation. Psychologically, phases can be seen as mental states (comp. The concept of “states of mind,” Horowitz, 1987), which are “applied to” or “enslaving” different topics. States can be induced by external or internal triggers. The phases and their specific subcategories are described below.

#### 3.1.1 Perceived pattern stability

All five patients repeatedly found themselves in reoccurring stable dynamics or attractors, which were indicated by persisting similar vocabulary. Stable attractors imply limited degrees of freedom (Kriz and von Schlippe, 2011). In the diary texts, this is expressed by the experience of lacking freedom of choice, the absence of alternatives, or automated cognitions, emotions, behavior, and subjective experiences. The term “problem” was frequently used to express suffering arising from the stability and rigidity of an attractor. Patients felt stuck in cognition-emotion-behavior patterns from which they wanted to escape. Phrases such as “I had to do…,” “I was forced to…,” or “I cannot help but…” were common in expressing this sense of constraint. Stability was perceived by three qualities (Table 3). The first one comprises statements illustrating that the patient feels pushed into a pattern, even by slight triggers. The second mirrors the pulling force of reduced degrees of freedom. A metaphor for this was a natural force, such as a fall or a violent suction into a pattern. The third quality was expressed by the feeling of being stuck.

**Table 3:** Three kinds of feeling overwhelming stability.

	Description	Examples
1	Minor triggers activated perceiving oneself as involuntary, externally controlled, and automated	Something produces reactions in me • minor events throw me off track
2a	Attractors are represented as a fall or suction	Carry away • sinking
2b	An attractor is perceived as a violent entity that exerts force on the person	I feel beaten down • something is pressing me
3	The attractor is presented as something the person is stuck in	I am stuck • something is holding me back

Stability and over-importance were also expressed by repeatedly mentioning the occurrence of a problem attractor, using phrases such as “as always” or “once again” combined with expressions of frustration and regret. During this phase, patients experience no distance from the problem, nor can they externalize it. However, they are aware of this and express a desire for sustainable changes in the system dynamics.

Within the category of perceived pattern (attractor) stability, three subcategories emerged.

##### 3.1.1.1 Feeling helpless in front of a problem attractor

If a person feels helpless in front of a problem attractor, they focus on the emotional impact of reduced degrees of freedom. Patients express a sense of vulnerability with phrases such as “I have nothing to oppose” or “I feel helpless.” In this phase, individuals often express feelings of hopelessness or frustration due to their inability to alter the problem. While frustration can be stimulating, hopelessness—if a patient does not manage to see any perspectives of improvement—may stabilize the problem and impede change.

##### 3.1.1.2 Noticing a problem attractor

When individuals notice a problem attractor, they approach it from a cognitive perspective, and it is only about its stability. Patients do not consider any alternatives, as in the subcategory of regaining control of the problem attractor.

##### 3.1.1.3 Regaining control

Gradually, patients can formulate strategies to gain control over behaviors and cognitions associated with problem attractors. Despite experiencing the stability of a problem attractor, they no longer feel entirely subject to its influence. Feelings of self-efficacy and helplessness may occur at the same time (ambiguity) or in sequence (critical instability). Patients may create positive experiences when trying to improve their situation. In the diaries, we find phrases such as “I oppose something” or “I fight against.” Noticing improvement of symptoms helps to get control. In some cases, the problem attractor feels less painful, or patients manage to reframe the meaning of the problem and accept it step-by-step.

#### 3.1.2 Noticing improvement in subjective experience and behavior

In this phase, patients report improvements in their subjective experience and behavior, which is contrasted against their problem attractors. The main indicator of this category, which comprises two subcategories, is its emphasis on creating contrast.

##### 3.1.2.1 Moment of improvement

The patient reports a single moment of improvement. Usually, this improvement is experienced as a personal achievement, and the patient feels proud of it. The moments are described by expressions such as “I managed to …” or “I succeeded in ….” Sometimes, changes also happen without specific achievement, such as noticing an increase in energy. Patients undergo the experience of behaving in contrast to their well-established problem attractor.

##### 3.1.2.2 Lasting improvement

When a patient experiences lasting improvement, improvement or pattern change is experienced as more or less stable. It is perceived as happening naturally or automatically, without considerable strain. No specific effort seems necessary to produce the improvement. This phase indicates a situation where a new adaptive attractor is emerging, perhaps also—as any attractor—reducing degrees of freedom, but now it is welcome.

#### 3.1.3 Broadening the perspective

In this phase, patients describe changing perspectives on a problem attractor and become aware of its variability. This is a movement into transformation. Patients describe the new perspectives as interesting, motivating, and encouraging. An energizing and stimulating effect is emphasized, which may catalyze further changes in cognitions, emotions, and behavior. Patients describe that they figure out explanations of how different aspects of a problem are interrelated and linked to each other. Indicators of broadening perspectives are expressions such as “I became aware of connections in this situation,” “something new revealed itself, that I was not aware of yet,” and “after the conversation I saw things differently.” In most cases, becoming aware of interconnections is experienced as energizing and motivating.

#### 3.1.4 Critical instability

During this phase, critical instabilities can be seen in the diaries. Patients articulate feelings of disorientation or confusion in their writing, describing a sense of losing direction or purpose. They express a state of instability, feeling off-balance, highly ambiguous, and convey a range of emotions with heightened intensity. The writing style is characterized by staccato, monosyllabic, vague, and scattered expressions. This is a moment without a stable and ordering attractor. The “gravitational force” of the previous stable attractor no longer has sufficient power. Loss of order can be overwhelming and induce anxiety in the future, but step-by-step, patients arrive to trust in the transformation process despite the uncertainty of its final state.

#### 3.1.5 Kairos

In this phase, diaries show that a patient is clearly in a process of transformation. They recognize that the moment of change has come and are ready for change. This may be seen as a “Kairos” moment—the time has come for change. Patients express an intuitive understanding of inner processes and describe autocatalytic effects, resulting in new stable dynamics attracting their attentional focus. New information gets in resonance and synchrony with the newly emerging attractor. Patients develop ideas for the future, and the focus of attention is directed to the outside world. This change in perspectives is often experienced as sudden, unplanned, and involuntary. Patients feel stimulated, inspired, and hopeful.

### 3.2 Case example 1

So far, we reported on general results regarding the phases of change developed based on the data from all five cases. The following two case studies will further concretize and illustrate these general results in detail. The surplus value of data triangulation—qualitative and quantitative analysis complementing each other—will become evident as it offers a more comprehensive and holistic understanding of the processes at hand.

The first patient (we call him “Stefan”) was a 31-year-old university student (a former case study focused on the time series; Schiepek et al., 2018b). He used the SNS for daily self-reports during his stay in a psychosomatic day-treatment center (79 days). Due to his introspective and self-reflective writing style, he cultivated a rational-cognitive attitude to comprehend his experiences and behavior. This approach allowed him to gain emotional distance from his experiences. On the one hand, the topics in his diaries focused on the symptoms of depression: fatigue and lack of energy, difficulties in maintaining a daily routine, and feelings of inner emptiness, as well as ruminations. On the other hand, he wrote about his problems with setting boundaries and standing up for himself in relationships with others. Stefan mentioned difficulties in expressing anger toward others and navigating conflicts. He observed that the moods or actions of others significantly influenced his own emotional state, leading to a disconnection from his own emotions. The recurrent topic in Stefan’s diaries is how to deal with his own emotions and his relationship with himself. This concerned his expression of sadness but also of anger, which he directed to himself instead.

**Figure 3:**
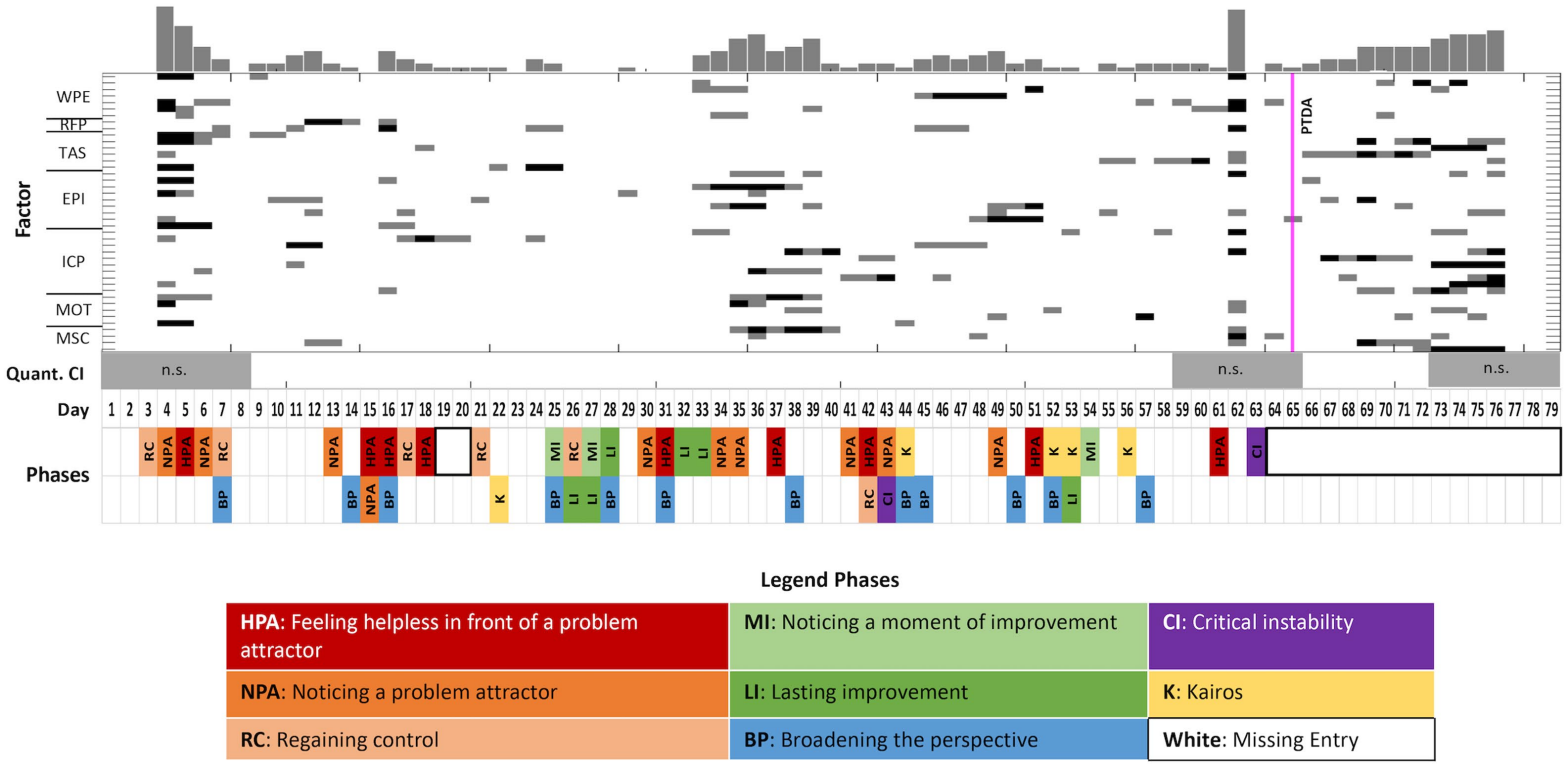
Case 1 (Stefan): A combined visualization of dynamic complexity patterns and the categories as identified by coding the diaries. Upper part: Complexity Resonance Diagram across all items related to the seven factors of the TPQ: “Well-being and positive emotions” (WPE), “Relationship with fellow patients” (RFP), “Therapeutic alliance and clinical setting” (TAS), “Emotional and problem intensity” (EPI), “insight/confidence/therapeutic progress” (ICP), “motivation for change” (MOT), and “mindfulness/self-care” (MSC). Gray pixels: The z-transformed complexity values exceed the 5% threshold of the confidence interval. Black pixels: The z-transformed complexity values exceed the 1% threshold of the confidence interval. The histogram on top of the diagram sums up all gray and black pixels. The pattern transition as it was identified by the PTDA is marked by the vertical magenta line. Lower part: Categories (resp. subcategories) as identified by the diary text analysis (comp. Figure 2). No diary entries are marked by white frames.

The PTDA indicated a pattern transition at day 65 with a probability of 0.74 (Figure 1, also marked in Figure 3 by the vertical magenta line). This was the only important phase transition prepared by a transient relapse. Figure 3 combines results from quantitative and qualitative data analysis. The upper part of the diagram (gray steps in the complexity resonance diagram) shows where, in the process, the dynamic complexity of each TPQ item is increased, and the lower part of the diagram illustrates the categories of the qualitative text analysis. The therapeutic process is stable during a long period of time (the first 64 days of the treatment). Although there were no indicators of transformation in the quantitative data before the transition, the analysis of the diaries indicated periods of stagnation and transformation also before the transition occurred.

During the first 3 weeks of his day-treatment stay, Stefan mostly perceived the stability of his problems. For example, on day 15, he felt stuck in a problem attractor. He was “somewhat confronted again” with struggling to set boundaries in encounters with others. He felt as if “something was stopping [him] from behind.” After day 22, 12 days in sequence referenced to transformation, starting with a Kairos. It was about the topic of “setting boundaries and standing up for myself.” He wrote: “As if it had been planned by someone, today in the psychodrama group I encountered exactly the topic that is currently occupying me anyway – setting boundaries, to notice when something bothers me. I want to change something, to signal STOP when someone crosses my personal boundary. Maybe I immediately reacted to this topic, because my perception is just a little focused in that direction right now – anyway, it does not really matter. In any case, this confrontation today, in combination with the experiences and thoughts of the previous days, has made me very confident to increasingly ‘understand’ something. This makes it possible for me to direct more attention in this direction and thereby to change something – or at least to become more aware of it.”

Up until that day, Stefan seldom mentioned the subject of setting boundaries and asserting himself. The first instance this topic arose was 6 days prior. Despite its infrequency, he described that this topic has been on his mind lately. He began the diary entry with the words “as if it had been planned by someone.” He was surprised since he had not expected this topic. In general, also for other patients, setting boundaries and standing up for oneself are central issues in psychotherapy, and in consequence, it is not unusual that issues such as this are treated in therapy groups. Therapists usually adapt the topics of a group session to the current needs of the patients, which Stefan was certainly aware of. Nevertheless, he felt it was a coincidence that this topic was discussed exactly that day. After the group session, he acknowledged this as an indicator of a process of pattern (trans-)formation and that the currently forming pattern autocatalytically influenced his attention. Furthermore, he saw that previous experiences in combination with this event now led to deepened comprehension. This may illustrate the non-linearity of change processes: First, different stimuli did not have any significant influence on his awareness (at least not big enough to report it in the diaries). Then, a threshold was reached at which the integration of insights could emerge. The therapeutic intervention (group discussion) occurred at the right time. Past experiences that were not directly related to this topic could have prepared this process. This made it “increasingly” evident that he was in a transformation process. By using quotation marks for the word “‘understand’” he expressed that he did not mean rational-cognitive understanding but rather an intuitive and affective understanding. Experiencing the process of pattern formation made him confident and had an energizing effect. In contrast to some previous diary entries on the topic of “setting boundaries and standing up for oneself,” which all had negative connotations, he now entered a Kairos. After this, he reported on this topic almost daily and now, the positive connotation of this topic has predominated the further sequence of diaries.

The experienced Kairos on day 22 was followed by phases of change. Stefan switched to a new medication on day 24, which may also have contributed to this. On days 25 to 28, he reported improvements in behavior, emotions, and subjective experience, which he perceived as lasting and which occurred in close connection with broadening his perspectives. Primarily, he reported on dealing with his own feelings.

Topics of broadening perspectives appeared frequently in periods of transformation but also occurred in times of stagnation. For example, during a period of stagnation between days 34 and 42, he experienced a moment of broadening perspectives at day 38 that gave him hope. In an encounter with fellow patients, he realized that—even if he wanted to—he could not express his aversion to their comments. In that situation, he became aware of the stability of his problem attractor. This awareness led to a change in perspectives on his own psychological field, which he had previously considered self-determined. Suddenly, he felt his position as an “imposed isolation” by which he punished himself for not standing up for himself. Interestingly, this insight had an energizing effect on him and improved his mood on that day.

A period of transformation started on day 43 (Figure 5). Day 53 was salient, where Stefan noticed a Kairos and lasting improvement. He had an impactful experience in a therapy session. He wrote: “In the individual therapy something ‘happened’ more than I did it grasp consciously, and yet I realized afterwards that this was a pretty big step for me. I somehow managed to put into words this feeling that I have been carrying around with me for ages (always) – the feeling that my existence is fundamentally not ‘okay’, ‘wanted’, or ‘good’. I do not know where this feeling comes from, but with the first almost ‘guileless’ utterance of this simple sentence (something like: ‘I have the feeling that it is not good that I am’) for the first time some kind of ‘acceptance’ of the feeling came along. No immediate jump to ‘This cannot be’ or ‘This is not a normal feeling, better put it aside and do not talk about it under any circumstances’ – of course not consciously, but felt somewhere. I have already talked about it in the past years and formulated it quite similarly. But always out of a different attitude, namely fighting against it and this ‘it must not be like this’. Only my theory about the whole thing, but I cannot explain otherwise why ‘it’ suddenly feels different. In the matter the same, but in the quality somehow different. Somehow very difficult to describe.”

**Figure 4:**
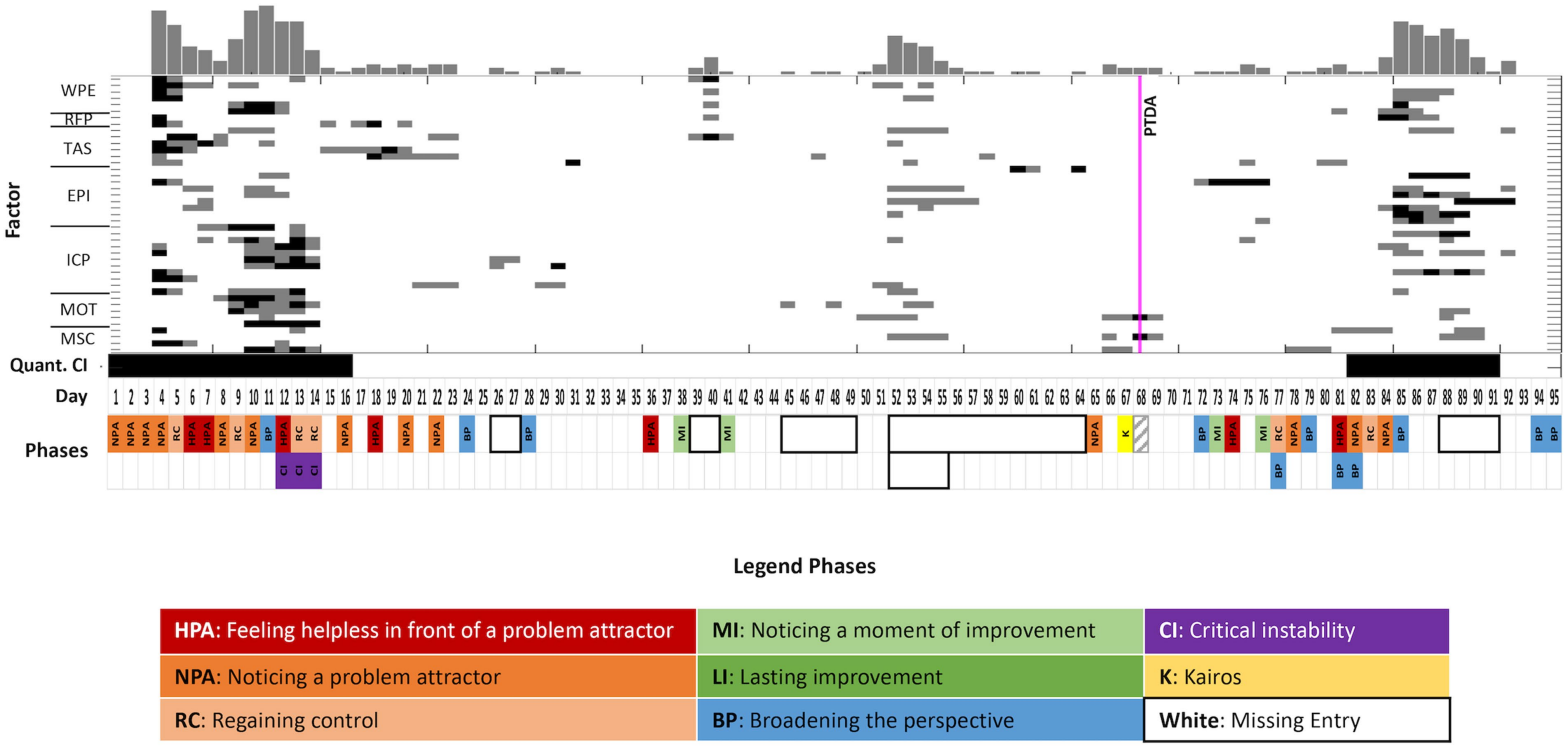
Case 2 (Martin): A combined visualization of dynamic complexity patterns and the categories as identified by coding the diaries. Upper part: Complexity Resonance Diagram across all items related to the seven factors of the TPQ: “Well-being and positive emotions” (WPE), “Relationship with fellow patients” (RFP), “Therapeutic alliance and clinical setting” (TAS), “Emotional and problem intensity” (EPI), “insight/confidence/therapeutic progress” (ICP), “motivation for change” (MOT), and “mindfulness/self-care” (MSC). Gray pixels: The z-transformed complexity values exceed the 5% threshold of the confidence interval. Black pixels: The z-transformed complexity values exceed the 1% threshold of the confidence interval. The histogram on top of the diagram sums up all gray and black pixels. The pattern transition as it was identified by the PTDA is marked by the vertical magenta line. Lower part: Categories (resp. subcategories) as identified by the diary text analysis (comp. Figure 2). No diary entries are marked by white frames.

**Figure 5:**
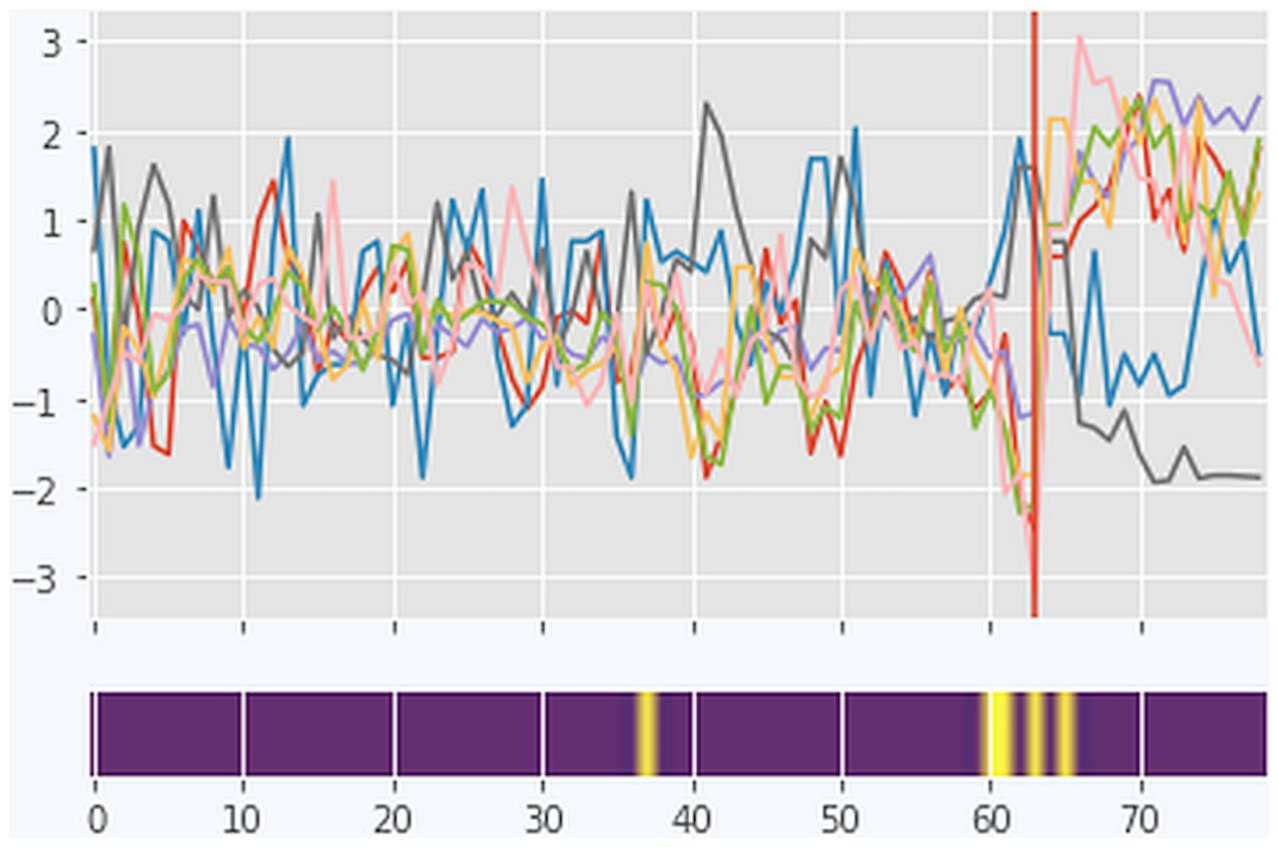
The pattern transition detection algorithm (PTDA) applied to the time series (factors of the TPQ) of case 1. Yellow color coding in the blue band represents the area with the highest probability of a pattern transition.

In the psychotherapy session he mentioned, Stefan experienced an unplanned and spontaneous “aha-moment,” which appeared automatically (“it simply ‘happened’”). He expressed a feeling that was accompanying and burdening him during his life. This feeling questioned his “raison d’etre” as a human being on an emotional level. Because of its fundamental nature, it represented a cornerstone on which he built his identity. Stefan reported that becoming aware of this feeling was not new, but it was the first time he was able to accept it and not judge it. He did not perceive this acceptance as a conscious decision but rather as something that was now automatically possible, as indicated by the vague term “a kind of ‘acceptance’.” He felt this not as a momentary success but as a sustainable change. He realized a qualitative change within himself (“In the matter the same, but in the quality somehow different”). Moreover, he felt it was a “big step,” which illustrated the non-linear jump of improvement. These are indicators that a new pattern was forming.

During the following days, this key moment was characterized by movement and energization. Then, 1 day after a phase of feeling enslaved by a problem attractor, on day 63, a phase of critical instability emerged in the diary entries. Stefan wrote: “Diffusely tired, unmotivated, as if ‘I had lost the thread’, great inner reluctance. Already addressed in individual therapy (the following day). Event in the mindfulness group, and only afterwards I became aware of its significance for me. I do not have the nerve to tell or describe it here now. But maybe I should make it a topic again.”

The entry on day 63 was the last one in Stefan’s diary, although he continued to fill in the TPQ for another 16 days until his discharge from the hospital, with a major change becoming evident in the time series. For the first 63 days, with two exceptions, Stefan wrote long entries every day, but then he stopped doing so after this phase of a transient relapse. Because there were no more diary entries, we have no information on how the transient relapse was experienced nor on how the new pattern was experienced, especially after the discharge. The quantitative data clearly show a phase transition to the better in almost all items and factors of the TPQ (Figure 5).

The analysis of the dynamic complexity in the time series did not yield a significant period of critical instability. Although there was an increased level of dynamic complexity during the period from day 59 to 65, this could not be distinguished from chance by surrogate data testing (gray bar at the bottom of Figure 3). The vertical line of bars just before the transition represents a small DC, indicating the transient relapse, which is mirrored by all items of the TPQ. The PTDA detects a pattern transition at day 65 (prob. = 0.74). There is a coincidence between the drastic change in the diaries (no further entries) and the subsequent phase transition. Subjectively experienced critical instabilities appear in the diaries just before the transition, but no significant DCs in the quantitative data. The period between days 52 and 57 is characterized by felt energy and transformation in the diaries and can be seen as a precursor of the phase transition. The emerging new attractor stimulated the transition.

The comparison of the qualitative and quantitative data showed that periods of transformation that could be identified in the diary entries did not always correspond to the quantitative data. This may be attributed to the different perspectives and subjective experiences when writing diaries or doing quantitative self-assessments. However, including and combining the two perspectives made it possible to fill informational gaps and clarify ambiguities, creating a holistic and detailed view of the case.

### 3.3 Case example 2

Martin is a 49-year-old teacher who filled in the TPQ for 95 days. He used the diary function regularly, but occasionally entries are missing. Martin frequently wrote about interactions with his ex-girlfriend and his son. At times, he provided detailed descriptions of emotions, but often, he limited himself to one-liners in which he linked different emotions using adjectives and nouns.

The topics in Martin’s diaries primarily revolved around his relationship with others. Particularly important were the relationships with his ex-girlfriend Carola, his son Lukas, and the son’s mother. Concerning these relationships, topics such as “self-worth,” “setting boundaries,” and “standing up for himself” were expressed. In the relationship with his ex-girlfriend, Martin felt dependent and guilty as soon as he tried to set boundaries. Martin repeatedly saw similarities to his own childhood. His doubts about “self-worth” corresponded to difficulties in taking responsibility and making decisions, which became salient in his relationship with his son and his son’s mother. In the relationship with Lukas’ mother, he experienced himself as a victim and passed the responsibility for his own behavior on to her. He also reported on conflicts with Lukas. “Lack of energy” was another important topic in the diaries, often associated with guilt and depressed mood.

During the first 2 weeks, Martin repeatedly alternated between feeling enslaved by a problem attractor concerning the relationship with his ex-girlfriend (e.g., day 6: “I’m kind of stuck in this feeling of dependency”) and regaining control by avoiding contact with her. These unstable dynamics peaked on days 12–14. On day 12, he became very emotional, expressing his despair and frustration. He wrote: “I’m crying, I’m just disappointed and angry how she’s dealing with me, how I’m letting her deal with me … how do I manage to break off contact and leave it at that, to hold firm …!????!”

As if this desperate moment of being stuck in the problem attractor would have been necessary to release the energy for it, Martin decided the next day: “In the evening I decided – I hope I stick to it!!!! – to break the contact with my ex-girlfriend, because she does not take me serious!” But just the next day, he struggled with this decision. Again, he expressed his anger and sadness. He also directed these emotions toward his parents because he attributed the origin of these stable dependency problems to his childhood. However, he regained control and ultimately kept his distance from Carola.

From the next day (day 15), both the topics and the emotional quality of the entries changed. The feeling of being dependent on his ex-girlfriend was replaced by feelings of guilt, while the turbulent emotionality of the previous entries on sadness and anger was combined with sorrow. The diary entries confirm a qualitative change at day 15, which was preceded by a phase of subjectively experienced critical instability, lasting 3 days, and by this, shorter than the increased DC of the time series data. The quantitative analysis of the DC yielded significant critical instability (*p* < 0.001) from days 1 to 16 (Figure 4). Corresponding to this, subjective ambiguity was experienced by Martin from the very beginning of his hospital stay, when he felt dependent on his ex-girlfriend or decided to break off contact.

The new attractor that started on day 15 was accompanied by painful emotions. On day 16, Martin identified recurring feelings of guilt as a problem attractor. He felt guilty for ending contact with his ex-girlfriend, yet he did not explicitly communicate this feeling. Instead, he reported on feelings of guilt out of context, suggesting that he perceived guilt as universal, not related to a specific person or occasion. During days 15 to 23, Martin wrote short entries, often with only one sentence. Lack of energy, depressed mood, and feelings of guilt were evident. On day 18, he again was enslaved by feelings of guilt.

On the following day, he was able to broaden his perspective. Martin suspected Carola to have a borderline personality disorder. He came across a YouTube channel on this topic that he found helpful. In the following 11 days, he perceived no stability in the problem pattern, which was different from the first 2 weeks and from diary entries until day 23. The YouTube channel seemed to be a resource to him, but in contrast to this, the therapeutic staff did not approve of his focus on the YouTube channel on borderline issues. Thus, on days 29 and 31, Martin reported feeling misunderstood by the staff. Emotions of shame and insecurity, as well as fear, sadness, and guilt, dominated his diary entries. The entries during this period were short and often consisted of strings of nouns or adjectives. The feeling of being misunderstood was accompanied by a lack of energy and a sense of sadness. After 13 days, the stable guilt attractor reoccurred again on day 36: “I am exhausted, and feel like being in front of a mountain of many conflicts/issues from my past ….”

However, on day 38, he noticed an improvement in his experience and behavior: “Today I felt better compared to the days before, particularly I had a little more time for myself.” On day 41, he noticed a further improvement related to success in setting boundaries. Martin thus experienced change, breaking free from the stagnant state characterized by a lack of energy, sadness, and guilt.

On day 44, he received a message from his ex-girlfriend on the death of a friend. This news affected him strongly, and consequently, during the next 18 days, he did not write any diary entries. Only on days 50 and 51, he wrote two short and very similar notes on guilt, lack of energy, and sadness. Evidently, he was not well during this time, and it can be assumed that the missing entries were an expression of a low energy level and depressive mood. The period of missing entries included the Christmas holidays. Not filling in the questionnaire was related to Martin’s decision to take time off from working on himself during the holidays.

On day 65, Martin resumed writing after he had filled in the TPQ regularly for 1 week. The next day, Martin again reported a situation where he felt like a victim: “I am accused to be a liar, which makes me uneasy and doubtful. But I also know that this ‘lie’ arose from an exceptional situation (fear of being rejected). Despite apologies and remorse, I am called a liar and subjected to emotional pressure.” He perceived unfair treatment, as he resorted to dishonesty out of fear of rejection. He found himself in a situation where he felt compelled to act in a certain way. Again, he felt stuck in a problem attractor, even if he did not realize this at that moment. He felt he had no choice and had already apologized and shown remorse. For him, the ‘lie’ was not a real lie and he did not feel like a real liar. He saw himself as a victim of manipulation. In this entry, he could not take another point of view.

**Figure 6:**
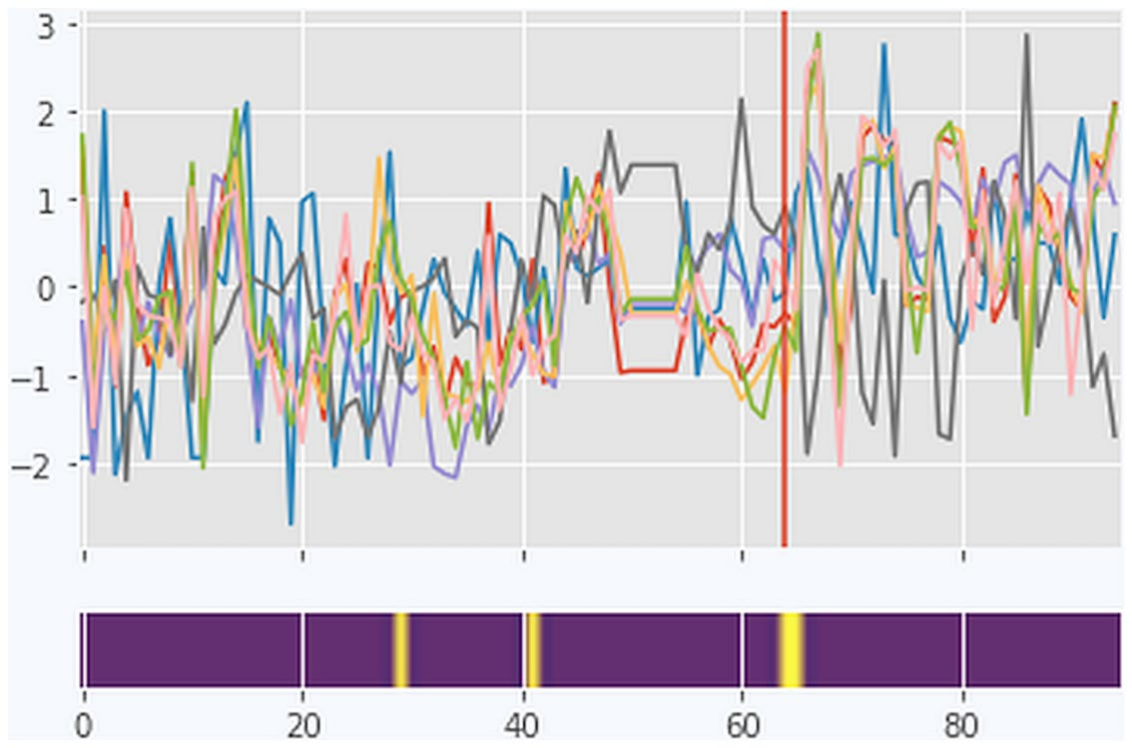
The pattern transition detection algorithm (PTDA) applied to the time series (factors of the TPQ) of case 2. Yellow color coding in the blue band represents the area with the highest probability of a pattern transition.

The following day, Martin noticed a Kairos moment in a psychotherapy session. He got instructions on how to see situations from an outside perspective, which he appreciated as “very interesting and positive!!!.” The entry expressed a high energy level, different from the previous entries, with a liberating quality: “I perceive the right direction in autonomy and self-esteem!.” He retrospectively gained a new perspective on the previous days. He noticed that he was in the middle of a promising process concerning his self-esteem and autonomy, and he recognized his ability to act and feel self-efficacious. The time series data reveal a phase transition detected by the PTDA 1 day after this phase of noticing a Kairos moment, at day 64 (prob. = 0.46, Figure 6). The pattern transition in the quantitative data can be directly related to the change in the diaries.

After the Kairos and the quantitative phase transition, Martin refrained from writing about his lack of energy and any feelings of guilt for the following 10 days. Even though he reported on conflicts with his son during the following days, he managed to respect his own boundaries in conflict situations (day 73). On day 75, he wrote about the relationship with Lukas, but he focused on meeting Lukas’ need for security instead of conflicts. He noticed improvement in experience and behavior on both days. On day 72, he reported a broadening of perspective when he realized that he had been excessively justifying himself in interactions with others, resulting in a loss of his own sense of self.

On day 77, Martin again was aware of the stability of his problems, concerned with his ex-girlfriend and experiencing feelings of guilt. However, this was combined with phases of broadening the perspective. Martin became aware of an internal conflict marked by ambiguity. On one hand, he grappled with the desire to keep his ex-girlfriend in mind, hopeful that she would initiate contact. On the other hand, he recognized that this interaction was harmful to his own wellbeing, leading him to consciously distance himself from seeking it. On day 81, Martin reported on a significant experience. When he hugged a young fellow patient, he perceived a disapproving look from his therapist. He was reminded of his childhood, inducing guilt for engaging in something forbidden—specifically, hugging an attractive woman. He was ashamed and afraid of being expelled from the hospital. After he found out that the critical gaze was not directed at him, he made himself aware of the conflict pattern (complex in the sense of analytical psychology) with the help of another therapist (broadening the perspective). He associated the assumed disapproving look to his mother since all topics of sexuality and intimacy were taboo within his family.

In the following days, Martin again perceived the stability of problem attractors. He perceived himself in the victim role with Lukas’ mother, carried away by the process of a specific conversation. However, prompted by a comment from an external counselor, Martin reflected on his own role in this situation. By recognizing that he had failed to take responsibility for his own actions and had instead viewed them solely as reactions to his son’s mother’s behavior, he was able to successfully broaden his perspective. He further extended this broadening of perspectives to his relationship with his ex-girlfriend. After writing about his perceived dependency (days 83 and 84), Martin realized that he had been unjustly blaming his ex-girlfriend for his own actions (day 85). In his reflection, he came to the realization that he had regarded his act of cheating through paid sex encounters as Carola’s responsibility. This perception was based on the belief that he had only engaged in such behavior due to feelings of inferiority toward his ex-girlfriend. He recognized his own responsibility for his actions. He reflected on his self-attributed victim role, in which he saw the other person as responsible for his own actions, taking the first steps toward taking responsibility for his own actions (day 87). This realization in relation to his ex-girlfriend led to further feelings of guilt about cheating. On day 91, he concluded the topic of his ex-girlfriend with the words, “Talked too much about my ex lately!.”

Quantitative analysis of dynamic complexity revealed significant critical instability (*p* < 0.001) during days 82 to 91 (Figure 4). The critical instability began 1 day after the significant experience of the fellow patient’s embrace, which triggered a broadening of perspectives concerning the pattern of intimacy. During the period of critical instability, a broadening of perspectives on his victim role followed. Martin repeatedly questioned his own behavior and actively changed his perspective on situations, and in consequence, he increasingly recognized his own responsibility and self-efficacy. The end of the critical instability period was flagged by a preliminary conclusion of the topic “ex-girlfriend” (day 91).

The last entry of his hospital stay documented once more a broadening of perspectives. In collaboration with a therapist, he realized that his constant urge to justify himself was related to feelings of guilt about being taken seriously and noticed. This last entry represented the final conclusion of his therapy and combined various topics and perspectives. For him, it was a starting point for an independent continuation of the change process.

To conclude, the change of topics on day 15 was heralded in the diaries by a period of psychological critical instability (ambiguity) and also critical instability in the quantitative data (from day 1 to 16). This corresponds to a transition period at the beginning of the treatment, which is common in many therapies. After this period, Martin’s focus changed, but he found himself in another problem attractor with feelings of guilt as an enslaving pattern. A pattern transition does not automatically lead to an improvement in well-being. The 18 missing diary entries between days 45 and 64 did not allow for a detailed analysis during this period but illustrated Martin’s lack of energy and depressive mood. In Martin’s diary, a Kairos was identified on day 67, when he recognized his progress so far and his self-efficacy through a sudden change of perspective, which had an energizing effect. This Kairos moment corresponded to a phase transition detected by the PTDA on day 64. From this moment, the lack of energy and feelings of guilt were no longer the main topics. The hug of a fellow patient (reported on day 81) made him aware of a problem attractor related to the taboo of sexuality and intimacy in his family. At this moment, the time series started a period of significant dynamic complexity. During this period, Martin questioned his self-attributed victim role and clarified his own responsibility for his actions (broadening of perspective). Martin perceived himself as still undergoing a process of change, now prepared to tread the path independently.

## 4 Discussion

Daily diary entries of five patients were examined to identify indicators of transformation and to compare these to quantitative features of time series data (daily sampling rate of self-assessments). Qualitative and quantitative data were analyzed by using the paradigm of self-organization. The indicators (categories) of transformation were developed within the Grounded Theory Methodology, with a focus on the way of reflection and communication (the “how” of processing) rather than on the content (the “what” of processing). From this perspective, the categories we developed are similar to the Innovative Moments Category System of Gonçalves et al. (2011), which evaluates therapeutic dialogues at a functional level rather than on a content level (Fernández-Navarro et al., 2020).

The transformation indicators were clustered under the key category of phases. In summary, there are phases of stagnation (e.g., “perceiving stability of a problem attractor”) and phases of transformation. Our classification followed the classification of innovative moments by Gonçalves et al. (2011, 2017) and Fernández-Navarro et al. (2020). For example, the phases “broadening the perspective” and “noticing a moment of improvement” are similar to the innovative moments “Reflection 1” and “Action 1,” which are phases of creating a mental distance to problems. The phases “noticing lasting improvement,” “critical instability,” and “Kairos” are phases that promote alternative meanings in a change process or describe improvements, with similarities to the innovative moments “Reflection 2,” “Action 2,” and “Reconceptualization.”

The phases we identified in the diaries emphasize the approach to handling various topics rather than the specific content. The patients applied similar process reflections to different topics, for example, when Martin reflected on his victim role in different relationships. The topics mainly provided the contents of the transformation processes, which were changed during phases of transformation in the diaries and in the time series data.

Indicators of transformation in the quantitative and qualitative data are not fully synchronized. This may be because the temporal resolution within a diary entry is finer, as it also maps fluctuations within a day. The identification of pattern transitions depends on sampling rates and on the applied measurement methods. Furthermore, quantitative and qualitative data introduce different perspectives on a psychological process. The subjective level of experience, as it is provided by diaries, complements the quantitative results and gives a subjective meaning and a psychological understanding of what is happening during critical instabilities or order transitions. Although empirical findings revealed a positive relationship between the intensity of critical instability and positive treatment outcome (Haken and Schiepek, 2010; Schiepek et al., 2014; Olthof et al., 2020a,b), the occurrence of order transitions not necessarily guarantees an improvement of well-being and should be specified by qualitative information.

### 4.1 Practical implications

The utilization of a mixed-methods design in this study adds meaning and allows sense-making to the results obtained from time series analysis and facilitates their interpretation, thereby enhancing their relevance for clinical practice. The SNS is an internet- and app-based digital tool for data collection and analysis (Schiepek et al., 2018a) available in clinical practice. It serves as a bridge between scientific research and practice, facilitating the integration of research findings into real-world clinical settings. It realizes the collection of time series and diary data during psychotherapy (Ochs et al., 2020). Repeated assessment of symptom severity of MDD is recommended during the psychotherapeutic process (Vittengl et al., 2014) and beyond MDD of all types of diagnoses (transdiagnostic). More than this, in SNS-based treatments, patients do self-assessments by using standardized or individualized questionnaires once per day. Schiepek G. et al. (2016) reported high compliance rates of frequent (daily) self-assessments with the SNS, regardless of diagnoses, symptom severity, or levels of stress. The crucial point was whether the procedure of monitoring made sense and seemed to be useful for the patients, and this also depended on the frequency and quality of feedback interviewing with the therapist(s).

Daily self-assessments help to structure the day and promote mindfulness and self-reflection on cognitions, emotions, and behavior, catalyzing the psychotherapeutic process (Schiepek et al., 2023). This corresponds to the findings of Suhr et al. (2017), who reported the positive therapeutic effects of writing daily diaries on depressive symptoms.

Time series and non-linear procedures of time series analysis implemented in the SNS are based on theoretical models of self-organization (Synergetics and chaos theory; Schiepek et al., 2018). Feedback from the SNS continuously keeps the theoretical foundation of the clinical work present and helps to concretize theoretical concepts by case-related data. Furthermore, therapists can enhance their confidence in individual change processes by leveraging the insights from this study. The recognition that existing topics and problem patterns persist and resurface throughout the process equips therapists with a greater sense of assurance during therapy.

### 4.2 Limitations

Indicators of transformation were developed on a sample of patients with MDD diagnosis (recurrent depressive disorder). This raises the question of to what extent these categories of transformation are specific to depression. Although the indicators are independent of specific topics, e.g., the experience of stability, which is associated with apathy and lack of energy, could be particularly pronounced in depression. Further investigation should be conducted in larger and more diverse samples to explore whether the experience of stability is related to other topics in healthy individuals or in individuals with different diagnoses.

This study was limited to five cases. Even if concepts with some explanatory power related to transformation processes could be extracted, the limited sample prevented the results from achieving theoretical saturation as required by grounded theory. Additionally, the data were mainly analyzed by a single individual (SN, psychologist), although facilitated by an ongoing interdisciplinary dialogue with a sociologist (PG). Nevertheless, there might be limitations in the inter-subjective reliability of the perspective on the data.

Sometimes, patients submitted entries about their daily experiences at a later time, resulting in a more retrospective perspective that was constrained by memory biases compared to writing the entry on the same day.

The sample exclusively comprised individuals who consistently filled out both the questionnaire and the diary function of the SNS. While this high commitment rate might pose a challenge for MDD patients, such as those experiencing a lack of energy, only those who successfully met these requirements were considered in this study.

### 4.3 Perspectives

Despite these limitations, this study shows the potential of mixed-methods designs within the paradigm of self-organization for psychotherapy research. Future research should develop indicators of transformation with patients of different diagnoses. Beyond diaries, other data sources such as interviews with patients, relatives, and therapists could be used, and by this, a perspective on interpersonal systems would be added. From the perspective of synergetics, a future focus on qualitative indicators for transformation might include control parameters, such as motivation for change.

Phases of stagnation (e.g., “perception of stability of a problem attractor”) occurred more frequently at the beginning of the change process, while phases of transformation occurred later. This tendency should be replicated by between-subject designs. Given the identification of correlations between innovative moments and positive therapy outcomes (Fernández-Navarro et al., 2018), there are potential correlations between the intensity and timing of indicators of transformation and treatment outcomes.

Artificial intelligence and machine learning present a completely different approach to the analysis of texts. These methods can be trained using existing text material to serve various purposes, such as the identification of categories in texts, the detection of precursors of specific events (e.g., pattern transitions), or the recognition of existing categories such as the ones we proposed in this study (Tausczik and Pennebaker, 2010). Irrespective of the specific goals and requirements, an advantage of these methods is their ability to process information rapidly in combination with their capability to integrate qualitative and quantitative analyses (Kolenik et al., under review). Conversational agents allow real-time interactions between the data-based pattern recognition of the AI agent and the professional (e.g., therapist) in natural language (Morris et al., 2018; Kolenik et al., under review).

## 5 Conclusion

This study contributes to understanding psychotherapeutic transformation processes from a perspective of self-organization. The qualitative analysis of diaries complemented results from quantitative time series analysis in the form of changing dynamic complexity (DC) and occurring pattern transitions. The combination of quantitative and qualitative results provided a detailed picture of individual transformation processes within the frame of self-organization theory. The study showed that qualitative analysis of patient diaries not only serves as a valuable foundation for further quantitative research but is also applicable in practical settings. Specifically, the qualitative results from this study offer a fertile ground for applying quantitative methods within the context of psychotherapeutic practice.

## Data availability statement

The data analyzed in this study is subject to the following licenses/restrictions: since the diary data contain extensive details about the patients’ lives we cannot make the datasets publicly available to protect their privacy. The time series data can be made available upon request. Requests to access these datasets should be directed to GS, guenter.schiepek@ccsys.de.

## Ethics statement

The studies involving humans were approved by the Ethics Commission Salzburg (Ethikkommission Land Salzburg, No. 415-E/1203/5–2012).The studies were conducted in accordance with the local legislation and institutional requirements. The participants provided their written informed consent to participate in this study. Detailed information on the study was provided and written informed consent was obtained from all participants according to the Declaration of Helsinki for the publication of any potentially identifiable images or data included in this article.

## Author contributions

SN: Data curation, Writing – review & editing, Writing – original draft, Visualization, Formal analysis, Conceptualization. PG: Writing – review & editing, Formal analysis. WA: Writing – review & editing, Visualization, Resources. MO: Writing – review & editing, Supervision, Data curation, Methodology. GS: Data curation, Methodology, Software, Writing – original draft, Writing – review & editing.
